# Team training in obstetric and neonatal emergencies using highly realistic simulation in Mexico: impact on process indicators

**DOI:** 10.1186/s12884-014-0367-1

**Published:** 2014-11-20

**Authors:** Dilys Walker, Susanna Cohen, Jimena Fritz, Marisela Olvera, Hector Lamadrid-Figueroa, Jessica Greenberg Cowan, Dolores Gonzalez Hernandez, Julia C Dettinger, Jenifer O Fahey

**Affiliations:** Department of Global Health, University of Washington, 325 9th Ave, Box 359931, Seattle, WA 98104 USA; Department of Obstetrics and Gynecology, University of Washington, 325 9th Ave, Box 359931, Seattle, WA 98104 USA; College of Nursing, University of Utah, 10 South 2000, East Salt Lake City, UT 84112 USA; Division of Reproductive Health, Research Center for Population Health, National Institute of Public Health, Universidad No 655 Col Santa Maria Ahuacatitlan, Cerrada los Pinos y Caminera, CP 621000 Cuernavaca, Mexico; Clinical Faculty, Department of Family Medicine, Swedish Hospital, 747 Broadway, Seattle, WA 98122 USA; Department of Obstetrics and Gynecology, University of Maryland School of Medicine, 22 S Green St, Baltimore, MD 12201 USA

**Keywords:** Interprofessional, Obstetric emergencies, Neonatal resuscitation, Simulation, Team training, Limited-resource

## Abstract

**Background:**

Ineffective management of obstetric emergencies contributes significantly to maternal and neonatal morbidity and mortality in Mexico. PRONTO (*Programa de Rescate Obstétrico y Neonatal: Tratamiento Óptimo y Oportuno*) is a highly-realistic, low-tech simulation-based obstetric and neonatal emergency training program. A pair-matched hospital-based controlled implementation trial was undertaken in three states in Mexico, with pre/post measurement of process indicators at intervention hospitals. This report assesses the impact of PRONTO simulation training on process indicators from the pre/post study design for process indicators.

**Methods:**

Data was collected in twelve intervention facilities on process indicators, including pre/post changes in knowledge and self-efficacy of obstetric emergencies and neonatal resuscitation, achievement of strategic planning goals established during training and changes in teamwork scores. Authors performed a longitudinal fixed-effects linear regression model to estimate changes in knowledge and self-efficacy and logistic regression to assess goal achievement.

**Results:**

A total of 450 professionals in interprofessional teams were trained. Significant increases in knowledge and self-efficacy were noted for both physicians and nurses (p <0.001- 0.009) in all domains. Teamwork scores improved and were maintained over a three month period. A mean of 58.8% strategic planning goals per team in each hospital were achieved. There was no association between high goal achievement and knowledge, self-efficacy, proportion of doctors or nurses in training, state, or teamwork score.

**Conclusions:**

These results suggest that PRONTO’s highly realistic, locally appropriate simulation and team training in maternal and neonatal emergency care may be a promising avenue for optimizing emergency response and improving quality of facility-based obstetric and neonatal care in resource-limited settings.

**Trial registration:**

NCT01477554

## Background

Worldwide in 2011 nearly 300,000 women died in pregnancy, childbirth and the postpartum period while there were approximately 3 million neonatal deaths and a similar number of stillbirths [1,2]. The vast majority of these deaths took place in low- and middle-income countries, where efforts have been made to improve rates of facility-based deliveries. Unfortunately, improved access to obstetric care has not consistently translated into better outcomes for women and infants [3,4]. Poor quality of institutional care, especially during emergencies, is a major contributor to maternal and neonatal mortality and morbidity [5,6].

Obstetric and neonatal emergencies are rare events. As a result, providers have few real-time opportunities to practice the necessary clinical, teamwork and communication skills shown to improve outcomes during such emergencies [7,8]. Traditional training approaches– including didactic sessions, manuals, and guidelines—have not been shown to improve adoption of evidence-based practice [7,9].

Simulation-based training in obstetric and neonatal care can introduce and reinforce evidence-based practices while improving communication and teamwork skills under realistic emergency conditions. The majority of such trainings are designed for and implemented in well-resourced countries utilizing costly simulation technologies [10,11]. Trainings created for developed country practice settings are unlikely to create sustainable change in limited-resource settings [12,13]. There is little research to date on using low-cost, low-tech yet highly realistic simulation strategies for quality improvement in middle and low income countries [14-17].

Over the last decades, Mexico has achieved widespread access to facility-based care with the majority (89.5% - 94.4%) of births attended by physicians [18]. Eighty percent of the more than 1200 maternal deaths reported in 2005 took place in hospitals, with more than 50% of these deaths attributable to common obstetric emergencies [11,18,19]. Therefore, Mexican hospitals located in regions with high rates of maternal and neonatal morbidity and mortality are an appropriate forum for a trial using simulation and team training to improve the quality of facility-based care.

This report describes a process indicator analysis from intervention hospitals in a recently completed pair-matched hospital-based control trial implementation trial evaluating the impact of a low-tech, highly-realistic simulation and team training program, *Programa de Rescate Obstétrico y Neonatal: Tratamiento Óptimo y Oportuno* (PRONTO) on maternal and neonatal outcomes. Outcome data from the implementation trial, including birth observations of normal deliveries and hospital-based neonatal mortality and maternal morbidity data, are in process and will be reported separately. For this report, changes in provider knowledge, self-efficacy, and teamwork were assessed in association with strategic goal achievement at the twelve intervention hospitals of the PRONTO implementation trial.

## Methods

### Development of the PRONTO approach

PRONTO is a highly-realistic, low-tech simulation-based obstetric and neonatal emergency training program that was initially developed for use in Mexico and is also currently being implemented in Mexico, Guatemala, and Kenya. A pilot study of PRONTO trainings, conducted between September 2009 and April 2010 in Mexico, demonstrated widespread acceptance of the training learning modalities and improved participant knowledge, self-efficacy, and teamwork [20].

The PRONTO curriculum is based upon World Health Organization standards in maternal and newborn care, Mexican national guidelines for obstetric care and best practices in the field of healthcare simulation [8,21]. Newborn resuscitation training is modeled after the American Academy of Pediatrics Neonatal Resuscitation Program (NRP) [22]. The teamwork and communication components represent the first adaptation of the TeamSTEPPS® team training program designed by the Agency for Healthcare Research and Quality to a middle- or low-income country setting [20,23]. The training has minimal didactic content; instead most teaching occurs through interactive team-building exercises, targeted skills sessions, highly realistic simulations of obstetric and neonatal emergencies, and video-guided debriefings immediately following each scenario. The videos of each simulation are used as the basis for objective-driven facilitated debriefing. Training sessions are led by an interprofessional team of nurse midwives, nurses and physicians including at least one PRONTO master trainer and 3–4 local team members that have completed a PRONTO train-the-trainer course.

A full PRONTO training consists of two modules, conducted two to three months apart. Module I is 16 hours in duration and conducted over two days while Module II requires 8 hours for completion and is conducted in one day. The focus of Module I is teamwork, obstetric hemorrhage, neonatal resuscitation and strategic planning. Module II builds on teamwork and communication concepts, reviews hemorrhage and neonatal resuscitation and introduces pre-eclampsia, eclampsia and shoulder dystocia training. The scenario topics selected were designed to reflect the common causes of obstetric and neonatal emergencies, including obstetric hemorrhage and preeclampsia which are the leading causes of maternal death in Mexico [24].

**Figure 1:**
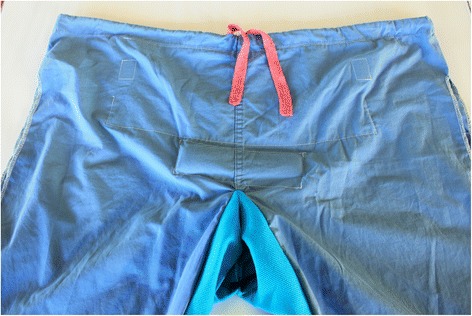
**The PartoPants™ are a low-cost, low-technology based birth simulator used for simulations during PRONTO International trainings.**

The simulations use a low-cost, low-tech hybrid birth simulator made by modifying recycled surgical scrubs (PartoPants™; PRONTO International, Seattle, WA, USA) (Figure 1). The pants have an opening to allow for birth, a pocket for simulated blood in an IV bag with tubing to simulate hemorrhage, a symphysis pubis for shoulder dystocia simulations, a simulated urethra to simulate catheterization and a simulated rectum to allow for administration of rectal misoprostol [25]. PartoPants™ are worn by a volunteer trainee who acts as the patient during the simulation, allowing for realistic provider-patient interactions. A cloth doll is used to simulate the neonate during delivery, while a NeoNatalie® mannequin is used for neonatal resuscitation simulation. Only the resources (e.g. medications, instruments, and staffing) that are normally available to healthcare teams within the facility are accessible during simulations. This provides physical and structural realism for the scenarios, ensuring that they are locally appropriate and helping to reveal practical supply, system and infrastructure gaps.

### Study design and measures

The PRONTO trial recently concluded in 24 hospitals in the states of Chiapas, Guerrero, and Mexico. These states were among the eight high-priority states identified by the Mexican Ministry of Health on the basis of high maternal mortality rates, and were selected because state ministries indicated interest in collaborating with PRONTO. We matched 24 hospitals meeting selection criteria based on volume of care, complication rates, infrastructure, medical equipment and medication resources and assigned them to the intervention (n = 12) or control group (n = 12). Baseline data collection started in June 2010; post-intervention data collection completed in March 2013. The intervention group received PRONTO trainings between August 2010 and January 2012, while control hospitals received no training. Using a pre/post-test design, we collected and analyzed process indicators from intervention hospitals.

Participation in PRONTO trainings at intervention sites was voluntary and offered to all physicians and nurses who worked directly with pregnant women or their infants during labor, birth or the postpartum period. Whenever possible, trainees conducted simulations with colleagues working on the same shifts within facilities. Participants played roles in simulation trainings based on their actual scope of practice, including cross-training as appropriate in personnel limited settings. Hospital staff participated in PRONTO trainings as part of their typical salaried duties, and no additional compensation was provided. All participants provided verbal informed consent to use written evaluations and simulation videos for research purposes. The Ethics and Research Committees at the National Institute of Public Health of Mexico provided approval on August 2, 2010 (Reference 845). The study is registered at clinical trials.gov: NCT01477554.

Process indicators collected in association with Module I and II trainings were as follows:

1. *Healthcare provider knowledge acquisition and self-efficacy.* Participants completed pre- and post-training questionnaires during both modules, evaluating knowledge of evidence-based practices in identifying, preventing, and managing obstetric and neonatal emergencies as well as participant confidence in his/her own ability to perform key skills (self-efficacy). The self-efficacy scales were based on the model of self-efficacy developed by Bandura [26]. The questionnaires were a revised version of those used in the 2010 PRONTO pilot [20]. Where applicable, we based questions upon the American Academy of Pediatrics Neonatal Resuscitation Program assessment tools [22].
  The pre- and post-training questionnaires included 138 items evaluating knowledge (50 questions) and self-efficacy (88 questions) in five categories: neonatal resuscitation, obstetric hemorrhage, general emergency, shoulder dystocia, and preeclampsia/eclampsia. In the case of the knowledge questions, participants’ responses were coded as dummy variables (0: incorrect, 1: correct), then we obtained a knowledge score (both total and for each category) consisting on the percentage of correct answers by each particular individual in the sample. In the case of self-efficacy items, the participants rated themselves on a scale of 0–100 in which 0 means complete lack of confidence and 100 means total confidence; in this case we defined the self-efficacy score as the arithmetic mean of the participant’s answers, both total and by category.
  Participants completed general obstetric emergency, obstetric hemorrhage and neonatal resuscitation knowledge and self-efficacy tests before and after Module I. Pre/post tests for Module II included knowledge and self-efficacy questionnaires for shoulder dystocia and pre-eclampsia. Additionally, following Module II, participants retook the self-efficacy questionnaire for general obstetric emergencies.
  We estimated the impact of the training on test scores by fitting a longitudinal fixed-effects linear regression model with fixed effects at the individual level, where the outcome variable was the test score of knowledge or self-efficacy and a dummy variable for time (before or after training) served as the main independent variable; the coefficient for this variable is the estimate of the impact of the training. The fixed-effects approach yields an estimate of the within-subject change in the outcome variables (knowledge and self-efficacy). Robust standard errors with clustering were calculated to take into account within-hospital correlation. Additional analyses included estimations of Pearson’s correlation coefficients to assess the correlation between self-efficacy and improved knowledge at baseline and follow-up for the sample as a whole as well as by profession.
2. *Teamwork performance.* PRONTO trainers assessed teamwork at 3 intervals, after day one of Module I (including 2 simulations, T1), after day two of Module I (including 6 simulations, T2), and after day one of Module II, (including 2 simulations, T3). Trainers used a scale based on the ten key behavioral skills for teams developed by the Center for Advanced Pediatric and Perinatal Education (CAPE) [27]. Trainers were not reminded of the previous ratings they gave to each team. The average within-hospital changes in teamwork performance after the trainings were estimated (and p-values obtained) by fitting a fixed-effects linear regression model with robust standard errors.
3. *Institutional goal achievement.* Since the majority of the simulations occur in-situ, using only available resources (human and material), experiences and events that occur during the simulated scenarios are used as the basis for a quality improvement process. At the end of the training, participants agree upon specific goals for practice or system change, with the aim of improving their facility’s maternal and perinatal care. Participants are asked to identify goals, outline the concrete steps for realizing these changes, assign individuals roles and identify outcome measures for each goal. The achievement of these goals is tracked 3-months later at the Module II training through a facilitated discussion in which successes, barriers to achievement, and follow-up measures are established. Mid-training reports with team goals are distributed to hospital administrators and government officials and the plan for follow at Module II is communicated to the leadership. There are no funds distributed for goal completion, as the objective of the strategic plan is for individual providers to begin to recognize their role and power in affecting change within a system and the importance of their participation in continuous quality improvement projects.
  Goals were categorized by researchers into three broad topics: teamwork, further training, or healthcare system changes. For analysis, goal achievement was defined as a dichotomous outcome variable identifying facilities with a greater than median proportional achievement of all goals. We performed logistic regression analysis to assess the correlates between high goal achieving facilities and greater than median improvements in knowledge, self-efficacy, and teamwork performance, proportion of physician or nurse participants, location (state) of facility, and participation by facility leadership.

## Results

A total of 450 participants underwent at least one module of the PRONTO training at the 12 intervention hospitals, with 305 completing both modules. Participants were physicians (54%) and nurses (46%). Between 6.4% and 31.6% of eligible medical personnel at each facility participated in Module I, with a mean participation rate of 20.5%. Module II had a mean participation rate of 15.2% (between 3.8 and 24.5% of all medical personnel). The facility inventory and baseline data collection indicated a range of patient volume, materials, resources, and personnel at intervention and control sites. All sites had ultrasound and some laboratory functions available (Table 1). Pre/post data for Module I and/or II is available for 450 participants.

**Table 1:** **Hospital and training participant characteristics for the 12 facilities receiving PRONTO Trainings**

**Participant characteristics (n =450)**	**Mean**	**sd**	**min**	**max**	**Facility infrastructure**	**Number (N =12)**
Age (years)	36.4	9.3	20.4	64.2	Blood transfusion capability	7
Males = 1 (vs Females = 0)	0.39^†^	-	0	1	Neonatal Intensive Care Unit	5
Physicians = 1 (vs Nurses = 0)	0.54^†^	-	0	1	Adult Intensive Care Unit	3
Morning Shift = 1 (vs Night Shift = 0)	0.40^†^	-	0	1	Laboratory	12
**Facility characteristics**	**Mean**	**sd**	**min**	**max**	Ultrasound	12
Total deliveries (previous year)	2495	1189.72	756	4613	Doppler	8
Mean distance to most used referral hospital (Km)	89.3	110.5	5	388	Uterine manual vaccuum aspiration capabilities	9
Percentage of personnel trained by PRONTO (%)	21	8	7	32	Obstetric emergency triage system	6
Number of all physicians	71.4	48.5	25	211	Oxytocin	11
Number of generalist physicians*	28.8	15.0	18	74	Misoprostol	3
Number of specialist doctors**	34.9	23.8	5	84	Magnesium Sulfate***	9
Total number of nurses	133.9	62.8	56	256		

*Generalists and interns.

**Obstetricians, Pediatricians, Anesthesiologist, Surgeons, Neonatologist, Perinatologist, Internists.

***N = 11 (one missing data).

^†^Proportion.

### Healthcare provider knowledge acquisition and self-efficacy

The results of pre- and post- training knowledge and self-efficacy assessments for management of obstetric hemorrhage, neonatal resuscitation, general obstetric emergencies, preeclampsia/eclampsia and shoulder dystocia demonstrate significant improvements (Table 2). There was improvement in both knowledge and self-efficacy for both physician and nurse participants across all categories. Physicians’ pre-test self-efficacy was higher (2.8 to 12.5 percentage points) than nurse measurements of pre-test self-efficacy, but nurse participants had significantly larger gains in post-test self-efficacy scores on most measures than did their physician counterparts. The correlation between self-efficacy scores and knowledge scores was uniformly low with pre-test Pearson coefficients for nurses ranging from -.07 to .07 in all subject areas and with post-test Pearson coefficients ranging from -.08 to .07 (Table 3). Physician correlations between self-efficacy and knowledge were somewhat stronger in the pre-test sample with Pearson’s coefficients ranging from .35-.13, decreasing in the post-test sample to .12-.05 (Table 3).

**Table 2:** Pre/post and change in knowledge and self-efficacy scores** by profession for management of obstetric and neonatal emergencies

**Variable**	**Overall**						**Doctors**						**Nurses**					
	**Pre**		**Post**		Change*	**(95% CI)**	**Pre**		**Post**		Change*	**(95% CI)**	**Pre**		**Post**		Change*	**(95% CI)**
**Knowledge**	**mean**	**sd**	**mean**	**sd**			**mean**	**sd**	**mean**	**sd**			**mean**	**sd**	**mean**	**sd**		
Obstetric hemorrhage	44.9	17.8	61.7	14.7	16.5	(11.95 - 21.07)	48.1	16.9	66.2	13.3	17.9	(14.20 - 21.55)	41.4	18.1	57.1	14.7	15.3	(9.20 - 21.34)
Shoulder dystocia (Module II)	52.2	24.5	68.4	20.1	16.0	(7.36 - 24.57)	54.7	23.4	73.2	18.5	18.5	(10.03 - 26.87)	49.9	25.3	63.9	20.5	13.5	(4.06 - 22.88)
Preeclampsia/Eclampsia (Module II)	54.3	18.1	69.0	16.5	14.6	(10.92 - 18.21)	60.8	16.6	72.9	15.8	12.5	(7.88 - 17.08)	48.3	17.4	65.6	16.5	16.5	(11–83 - 21.17)
Neonatal resuscitation	51	19.9	66.2	18.6	15.4	(11.23 - 19.63)	55.7	18.7	71.8	17.8	16.4	(11.91 - 20.92)	46.1	20.0	60.7	17.7	14.5	(9.35 - 19.54)
**Self-efficacy**																		
Obstetric hemorrhage	73.6	19.5	90.4	10.6	17.1	(13.61 - 20.66)	78.7	17.0	94.7	5.8	15.5	(12.83 - 18.11)	68.1	20.5	86.5	11.9	19.0	(13.75 - 24.30)
Shoulder dystocia (Module II)	67.2	24.4	90.6	11.4	23.9	(18.70 - 29.04)	73.1	23.5	93.6	8.8	20.9	(14.25 - 27.58)	62	23.5	88.4	12.2	26.4	(21.06 - 31.82)
Preeclampsia/Eclampsia (Module II)	84.6	12.5	93.6	8.0	9.4	(7.92 - 10.87)	87.9	10.4	95.5	6.4	7.9	(5.12 - 10.71)	81.5	13.6	92	8.9	10.8	(9.52 - 12.07)
Neonatal resuscitation	80.4	15.6	94.0	7.7	13.9	(11.95 - 15.83)	82.1	14.1	96.1	5.7	13.9	(11.24 - 16.56)	78.6	17.0	92.1	8.7	14.0	(10.50 - 17.40)
General Obstetric Emergency; Module I	83.1	14.4	93.2	8.3	10.5	(8.44 - 12.58)	84.6	13.2	94.2	6.9	9.8	(7.93 - 11.59)	81.6	15.5	92.7	8.0	11.5	(8.48 - 14.59)
General Obstetric Emergency; Module II	87.2	12.1	93.7	8.2	6.7	(4.480 - 9.01)	88.8	11.7	95.3	6.2	6.7	(4.25 - 9.23)	86	11.2	92.5	9.0	6.6	(4.05 - 9.08)

*Fixed effects estimator (clustering at hospital level), by profession.

**Minimum and maximum possible values: 0–100.

**Table 3:** **Pre/post training pearson’s coefficients (r) of correlation between knowledge and self-efficacy by theme and profession**

**Overall**	**Pre**		**Post**	
	**r**	**p**	**r**	**p**
Attention to newborn	0.16	0	0.14	0.01
Obstetric hemorrhage	0.09	0.09	0.15	0.01
Shoulder dystocia	0.12	0.03	0.03	0.59
Preeclampsia/ Eclampsia	0.18	0	0.09	0.13
**Nurses**	**Pre**		**Post**	
	**r**	**p**	**r**	**p**
Attention to newborn	0.02	0.78	0.07	0.37
Obstetric hemorrhage	−0.05	0.54	0.03	0.68
Shoulder dysstocia	0.07	0.41	−0.08	0.3
Preeclampsia/Eclampsia	−0.07	0.38	0.04	0.67
**Doctors**	**Pre**		**Post**	
	**r**	**p**	**r**	**p**
Attention to newborn	0.26	0	0.05	0.52
Obstetric hemorrhage	0.14	0.05	0.11	0.17
Shoulder dysstocia	0.13	0.13	0.12	0.19
Preeclampsia/ Eclampsia	0.35	<.001	0.07	0.42

### Teamwork performance

There was significant teamwork improvement in all areas between the Module I, day 1 (T1) and Module I, day 2 (T2) as well as significant improvement between Module 1, day 1 (T1) and Module 2 (T3), which took place approximately 3 months later (Table 4).

**Table 4:** **Changes in teamwork assessment on a 1 (poor) to 10 (excellent) scale over time**

	**Stage**						**Comparison**					
Variables**	**T1**		**T2**		**T3**		**T1 vs. T2**		**T1 vs. T3**		**T2 vs. T3**	
	**mean**	**sd**	**mean**	**sd**	**mean**	**sd**	**β**	**95% CI**	**β**	**95% CI**	**β**	**95% CI**
**Overall teamwork score**	3.90	0.70	6.68	0.68	6.94	0.78	2.76*	(2.25, 3.27)	2.99*	(2.49, 3.49)	0.23	(−0.25, 0.71)
**Thinks out loud**	3.35	0.88	7.04	0.96	6.79	1.10	3.68*	(2.99, 4.36)	3.43*	(2.70, 4.11)	−0.25	(−0.89, 0.39)
**Uses clear and directed communication**	2.95	0.64	6.54	0.78	6.73	1.00	3.59*	(2.89, 4.28)	3.77*	(3.08, 4.47)	0.18	(−0.47, 0.85)
**Knows their environment/anticipates the situation.**	4.45	1.04	6.58	0.70	6.96	0.99	2.10*	(1.36, 2.83)	2.47*	(1.74, 3.20)	0.37	(−0.32, 1.07)
**Optimal use of human and material resources**	4.15	1.27	7.00	1.02	6.96	0.96	2.84*	(1.94, 3.74)	2.8*	(1.90, 3.70)	−0.04	(−0.90, 0.81)
**Uses all information to develop and action plan**	3.35	1.13	7.08	1.08	6.40	1.09	3.70*	(2.83, 4.57)	3.02*	(2.14, 3.89)	−0.68	(−1.51, 0.14)
**Roles are well defined**	4.15	1.49	6.41	0.97	7.71	0.66	2.22*	(1.41, 3.04)	3.51*	(2.71, 4.30)	1.28*	(0.51, 2.05)
**Leadership**	2.65	1.03	5.92	0.73	6.92	0.93	3.26*	(2.51, 4.01)	4.26*	(3.51, 5.01)	1	(0.28, 1.71)
**Distribution of tasks/delegation of activities/mutual support**	3.45	1.42	6.50	1.33	6.94	1.60	2.93*	(1.98, 3.88)	3.37*	(2.41, 4.32)	0.43	(−0.45, 1.33)
**Provision of adequate care to patient and family**	3.90	1.52	6.42	1.65	6.75	1.52	2.39*	(1.77, 3.01)	2.73*	(2.11, 3.35)	0.33	(−0.24, 0.91)

**Based on ten key behavioral skills for teams by the Center for Advanced Pediatric and Perinatal Education.

*p- value <0.001.

T1 = Time 1 = End of day one of training Module I.

T2 = Time 2 = End of day two, Module I.

T3 = Time 3 = End of day 3, Module II.

### Institutional goal achievement

The 12 intervention hospitals together identified 124 goals of which 33 focused on teamwork, 35 focused on additional training and 56 focused on system changes. After a three-month interval, between 2 and 12 goals were achieved by participant teams (mean = 6 goals) at each site. 73 (58.8%) of these goals had been completed including 28 (80%) of training goals, 30 (53%) of system change goals and 15 (45%) teamwork goals (Table 5). While healthcare provider teams identified health system oriented goals more often than teamwork or training related changes, training related changes were achieved at a much higher rate. See Table 4 for examples of institutional goals that were achieved and not achieved.

**Table 5:** **Overview of goals and achievement rates in three categories: training, system and infrastructure, and teamwork**

**Category**	**Number of goals identified (124 total)**	**Number of goals achieved (73 goals, 58.8%)**	**Examples of goals achieved**	**Examples of goals not achieved**
Training	35	28 (80%)	Replicate simulation scenarios with PartoPants™	Train in manual uterine vacuum aspiration
			Train other personnel in Active Management of the Third Stage of Labor	Improve record keeping for charts
			Train additional personnel in teamwork concepts	
			Train additional personnel in communication rules	
System and Infrastructure	56	30 (53%)	Implement an alarm system useful throughout the hospital, emergency department, labor and delivery, pediatrics	Acquire new ambu bags for neonatal resuscitation
			Acquire medications such as Misoprostol, Oxytocin	Establish meetings between hospital Director and local government health authorities
			Refrigeration of oxytocin and/or ergonovine	Access to locked ultrasound machine evening and weekends
			Move refrigerator close to delivery room	Reorganize shift coverage to make sure adequate care available
			Create inventory of available medications for obstetric emergencies	Specialty care available in all shifts
			Repair ambulance	
Teamwork and Communication	33	15 (45%)	Post and promote characteristics of a strong leader	Improve work environment through courses and seminars
			Use communication rules with colleagues and explain their use to non-PRONTO trained colleagues	Establish system of individual stimuli for better attitude and teamwork- “provider of the month”
			Meeting between PRONTO participants and hospital authorities, Medical and Nursing Directors, to discuss action plan and implementation	

We performed a hospital-level logistic regression analysis to assess whether pre or post-test knowledge and self-efficacy scores, teamwork assessments, percentage of hospital personal attending the courses, and facility leaders attending the course, were predictive of facilities achieving an absolute number of completed goals greater than the median (median = 4.5 goals). The analysis was done fitting both simple and multiple (multivariate) models. Although neither factor was significantly associated to goal achievement, the odds of having above-median goal achievement were much higher in those hospitals with high self-efficacy scores (OR = 4 and OR = 10.55 for the simple and multivariate model, respectively) (Table 6).

**Table 6:** **Logistic regression analysis of variables predictive of above median goal achievement**

	**Simple analysis**		**Multiple analysis**	
**Variable** ^**†**^	**Odds ratio**	**p-value**	**Odds ratio**	**p-value**
**Average knowledge (1 = high, 0 = low)**	1.02	0.86	1.68	0.72
**Average self efficacy (1 = high, 0 = low)**	4.00	0.26	10.55	0.17
**Percentage of doctors attending PRONTO course (1 = high, 0 = low)**	1.00	1.00	1.13	0.94
**Percentage of nurses attending PRONTO course (1 = high, 0 = low)**	1.00	1.00	2.52	0.61
**Average teamwork performance (1 = high, 0 = low)**	1.42	0.70	2.85	0.55
**State**				
**State of Mexico (reference)**	1.00	1.00		
**Chiapas**	1.00	1.00	--	--
**Guerrero**	1.00	1.00	--	--

^†^Logistic regression analysis: All variables dichotomized at median, except state.

## Discussion

### Main findings

Post-training results indicate that knowledge of evidence-based practice in the management of obstetric emergency and neonatal resuscitation markedly improved for all providers between pre- and post-tests. These data also suggest that inter-professional simulation training improves self-efficacy measures among both doctors and nurses. Interestingly, markers of self-efficacy improved notably for nurse participants but showed less change for physician participants. Improved self-efficacy may correlate with enhanced confidence in identifying and managing maternal emergencies. Differing levels of self-efficacy between provider groups may alter team dynamics.

The poor correlation between knowledge and self-efficacy, however, raises questions regarding participants’ understanding of personal knowledge gaps. This poor correlation may suggest that participants reported improved self-efficacy in some areas because of the expectation for improvement, a form of social desirability bias. PRONTO training presents the latest recommendations for evidence-based care, and for many participants the concepts presented represented new knowledge and ultimately will require a long-term change in practice. This presentation of new knowledge coupled with the tendency of simulation training to highlight potential errors or knowledge gaps could have affected post-test self-efficacy, especially in physicians. Alternately, physicians may have demonstrated smaller gains in self-efficacy than nurses because of over-estimation of professional abilities before the trainings.

Teamwork measurements also suggested that team coordination and use of specific communication techniques improved over the course of the training, with a large initial increase, followed by a sustained improvement that was shown at in Module II, although at levels lower than those demonstrated at the conclusion of Module 1. This attrition of skills suggests that change in communication habits is a long, iterative process that needs reinforcing. However, the teams did not return to baseline, suggesting that new skills were integrated to some extent. The teamwork scale used for this report represents a subjective measure of teamwork that was based on validated teamwork measures however the tool itself not validated officially by the research team, other than for face validity by expert team trainers [27]. This change in teamwork points to the need for more precise teamwork coding and rating through the use of video analysis, a project currently underway by the research team.

Institutional goal achievement at three-month follow-up was encouraging. This outcome suggests that simulation may serve as an effective foundation for team-based quality improvement, as goals identified by hospital teams were strongly influenced by gaps and challenges that emerged in the setting of simulation scenarios. Though the logistic regression analysis is limited due to the small sample size, the non-significant association between self-efficacy and goal achievements is worth highlighting. It may indicate synergy between PRONTO training, improved self-efficacy and goal achievement, which together with increased knowledge may lead to more coordinated emergency response and improved outcomes.

There is tremendous urgency to improve the quality of facility-based obstetric and neonatal care in many low and middle-income countries. Though evidence exists for the value of simulation for obstetric emergency training in high-resource settings, there is limited evidence supporting use of low-tech models, particularly in limited-resource settings [14,15,28]. Most peer-reviewed publications describing training in emergency obstetric care in limited-resource environments do not include simulation and have evaluated only post-training knowledge in small numbers of trainees. A recent review of simulation for maternity care identified only one article evaluating impacts of simulation-based training in a limited-resource setting [12].

The process results presented in this report are a proxy for change in clinical practice by providers and teams and provide encouraging evidence for the acceptability and applicability highly-realistic, locally appropriate inter-professional simulation training for maternal and neonatal care in Mexico using the PRONTO model. Our results will be used to improve the quality of PRONTO trainings. Future research should include analyses of how these changes in individual and system level process indicators impact change in behavior both through birth observations and hospital-level outcome data.

### Strengths and limitations

This study is strengthened by the number of participants and sites included in the analysis. A recent systematic review of in-service training in emergency obstetric care in limited-resource environments identified only 38 papers for inclusion; 37 of the included trainings included fewer than 150 skilled birth attendants [29]. Small-scale evaluations of in-service trainings in maternal care have limited power to demonstrate efficacy or effectiveness in changing provider knowledge and behavior, team function and maternal and neonatal outcomes. With the inclusion of 450 participants, the sample size is large enough to measure the effect of the PRONTO training on participant self-efficacy and knowledge, teamwork, and strategic goal achievement. This paper discusses process indicators evaluated within the framework of a controlled trial designed to measure neonatal and maternal outcomes; outcome data is currently being analyzed and will be discussed in a subsequent publication. The larger trial’s quasi-randomized controlled design will ultimately strengthen the evaluation of causality of impact results though no control data is available to evaluate changes in process indicators.

The study has a number of limitations to consider. First, teamwork assessment was based on subjective scoring by facilitators who had a professional investment in improving team dynamics. Future analysis of simulation videos will employ an objective scoring of teamwork that was beyond the scope and resources of this paper. Given the intensive nature of a simulation-based training program, PRONTO training groups are limited to 20–30 participants per training. Even with two training sessions at larger institutions, we were only able to train an average of 21% of providers working in maternal and child health in the facility. Future roll-out will include more time and funding to ensure a higher proportion of facility staff is reached.

### Interpretation

This study addresses both applicability and effectiveness of PRONTO training to change provider-based outcomes across a relatively large sample of facilities and maternal and neonatal healthcare providers. PRONTO trainings, which were conducted in 12 hospitals in three high priority Mexican states, increased provider self-efficacy and knowledge scores, and promoted facility-level obstetric care process, infrastructure and teamwork improvements. High levels of facility-based change were accomplished despite relatively low total percent of personnel trained; suggesting that even partial participation in combined simulation and team trainings at training sites can result in progress. While these improved process indicators are promising, the obstetric safety literature has yet to convincingly demonstrate a correlation between process indicators such as knowledge, self-efficacy and teamwork and changes in neonatal and maternal outcomes.

## Conclusions

The tools and materials used in PRONTO’s simulation based training are well within the reach for both low- and middle-income countries, which currently invest in primarily didactic training programs that carry little evidence documenting significant impact. PRONTO’s results suggests that high fidelity simulation and team training in combined maternal/neonatal care may be a promising avenue for improvement in provider knowledge and promotion of facility-based quality improvements in resource-limited settings.

## Abbreviations

PRONTO: Programa de Rescate Obstétrico y Neonatal: Tratamiento Óptimo y Oportuno
NRP: Neonatal Resuscitation Program
CAPE: Center for Advanced Pediatric and Perinatal Education
